# Bright days, dark nights: translating science on the non-visual effects of light into public health guidance

**DOI:** 10.3389/phrs.2026.1609683

**Published:** 2026-07-07

**Authors:** Maya Piper, Kai Broszio, Jan Denneman, Marijke Gordijn, Elise McGlashan, Renske Lok, Raymond P. Najjar, Luke Price, David Sliney, Oliver Stefani, Juliette van Duijnhoven, Laura Kervezee, Manuel Spitschan

**Affiliations:** 1 Light for Public Health Initiative, Munich, Germany; 2 Federal Institute for Occupational Safety and Health (BAuA), Dortmund, Germany; 3 Good Light Group, Eindhoven, Netherlands; 4 Chrono@Work, Groningen, Netherlands; 5 Groningen Institute for Evolutionary Life Sciences, University of Groningen, Groningen, Netherlands; 6 Melbourne School of Psychological Sciences, The University of Melbourne, Parkville, VIC, Australia; 7 Department of Integrative Physiology, University of Colorado Boulder, Boulder, CO, United States; 8 Department of Ophthalmology, Yong Loo Lin School of Medicine, National University of Singapore, Singapore, Singapore; 9 Singapore Eye Research Institute, Singapore, Singapore; 10 UK Health Security Agency, London, United Kingdom; 11 Bloomberg School of Public Health, Johns Hopkins University, Baltimore, MA, United States; 12 Lucerne University of Applied Sciences and Arts, Lucerne, Switzerland; 13 Department of the Built Environment, Building Lighting group, Eindhoven University of Technology, Eindhoven, Netherlands; 14 Department of Cell and Chemical Biology, Leiden University Medical Center, Leiden, Netherlands; 15 Max Planck Institute for Biological Cybernetics, Translational Sensory & Circadian Neuroscience, Tübingen, Germany; 16 TUM School of Medicine and Health, Technical University of Munich, Munich, Germany; 17 TUM Institute for Advanced Study (TUM-IAS), Technical University of Munich, Garching, Germany; 18 TUMCREATE Ltd., Singapore, Singapore

**Keywords:** chronobiology, circadian rhythm, consensus statements, daylight, health guidelines

## Abstract

**Background:**

Light is a daily environmental exposure that can influence circadian timing, sleep, alertness, mood, and cognition, yet public health guidance still focuses mainly on optical-radiation hazards rather than healthy patterns of visible light and darkness.

**Analysis:**

This policy brief translates recent evidence and expert consensus on the non-visual effects of ocular light exposure into public health guidance. The evidence supports a practical 24-h pattern: brighter light during the morning and daytime, lower light in the evening, and darkness or near-darkness during sleep. This principle should be communicated as general guidance rather than a universal dose, because responses vary with timing, intensity, spectrum, duration, prior light exposure, age, work schedules, health status, and geography.

**Policy options:**

Public health organizations can communicate plain-language guidance, integrate light into workplaces, schools, healthcare, elder care, housing, and public buildings, align daylight messages with sun-safety advice, and support research, measurement, and melanopic labeling.

**Conclusion:**

Clear, proportionate guidance on light and darkness can help people and institutions structure daily environments to support circadian alignment, sleep, alertness, mental health, and wellbeing.

## Background

Light is a public health exposure because it is encountered by nearly everyone every day and because the timing, intensity, spectrum, duration, and spatial distribution of light reaching the eye can influence physiology and behavior [1, 2]. This policy brief addresses the non-visual effects of ocular light exposure: effects mediated through the eye that are not primarily about forming images, including effects on circadian timing, sleep, alertness, mood, and cognition.

The central policy problem is that modern daily light exposure often differs from the pattern to which the human circadian system is adapted [3]. Many people spend most of the day indoors, where light levels are often much lower than outdoors, and are exposed to electric lighting and self-luminous screens in the evening and at night [4–6]. Public health guidance has traditionally emphasized hazards of excessive optical radiation, such as ultraviolet exposure, but has provided less systematic advice on how the daily pattern of visible light and darkness can support circadian health.

The aim of this brief is to help public health organizations translate the current science into practical, proportionate, and non-alarmist guidance for the general population. The target audience is public health agencies and adjacent organizations that develop health communication, occupational guidance, school and care-setting recommendations, and built-environment advice. The brief is not intended to replace clinical guidance, lighting design standards, or individualized treatment for sleep, circadian, psychiatric, ophthalmic, or occupational health conditions.

The added value of this document is translational. The *Light for Public Health Consortium* previously developed 26 plain-language consensus statements on visible light, human photoreceptors, and non-visual responses to light [7]. This policy brief does not duplicate those statements but reorganizes the evidence around public health action, defines key terms, operationalizes common messaging phrases, identifies implementation limits, and sets out policy options that organizations can adapt for communication and policy development.

## Analysis

### Research evidence

The discovery and characterization of intrinsically photosensitive retinal ganglion cells (ipRGCs) provided a biological basis for understanding how light can affect physiology beyond vision [8]. These retinal neurons contain melanopsin and also receive input from rods and cones. They project to brain areas involved in circadian timing and other non-visual responses. A key output is the regulation of melatonin, a hormone that normally rises in the evening and signals biological night.

The human circadian system is not a single rhythm. It is a coordinated timing system that includes the central circadian pacemaker and rhythms across many tissues and behaviors. In this brief, circadian rhythm refers to a measurable near-24-h rhythm, such as the rhythm of melatonin or sleep timing. Circadian alignment refers to the appropriate temporal relationship between internal rhythms, behavior, and the external day-night cycle. Circadian misalignment or circadian disruption refers to a mismatch or disturbance in these relationships, for example, when light exposure, sleep, work, or meals occur at times that conflict with biological night [1].

Light can shift the timing of circadian rhythms. A phase advance means that a rhythm occurs earlier, for example, an earlier onset of evening melatonin or an earlier preferred bedtime. A phase delay means that a rhythm occurs later. In simplified terms, light in the morning tends to advance circadian timing, whereas light in the evening and early night tends to delay it. The size and direction of the effect depend on timing, intensity, spectrum, duration, prior light history, and individual sensitivity.

The evidence base supports a general population message: aim for relatively bright light during the morning and daytime, reduce light exposure during the evening, and keep the sleep environment dark or near-dark at night. This pattern is expected to support circadian alignment and may support sleep, mood, alertness, and cognition [1, 2]. The recommendation should be communicated as a directional principle rather than a precise dose for every person.

Several lines of evidence are relevant for public health messaging. Brighter daytime light is associated with better sleep timing and sleep quality in many settings. Bright light can acutely increase alertness and cognitive performance, although findings depend on task, time of day, prior light exposure, and light characteristics [9–11]. Evening and nighttime light can suppress melatonin, delay circadian timing, and affect sleep, particularly when it is bright, short-wavelength enriched, close to the eyes, or viewed for prolonged periods [12].

### Operational definitions for public guidance

Terms such as “healthy light exposure” “right light” and “bright light” can be useful in public communication, but they should be defined operationally. In this brief, healthy daily light exposure means a 24-h pattern of ocular light exposure that is likely to support circadian alignment and sleep-wake regulation for most people: brighter light in the morning and daytime, dimmer light in the evening, and darkness or near-darkness during sleep. It does not imply that there is one universally correct exposure pattern or that more light is always better.

Bright light means light that is strong enough at the eye to have a meaningful non-visual effect, given its timing, spectrum, duration, and direction. For technical communication, the most relevant metric is melanopic equivalent daylight illuminance, or melanopic EDI, which is measured in lx (lux) and is designed to quantify stimulation of the melanopsin-containing ipRGC pathway [8, 13]. Expert recommendations for healthy adults propose indoor melanopic EDI above 250 lx during the day, below 10 lx in the evening, and below 1 lx during sleep [2]. However, these values are difficult for most people to apply because consumer products rarely provide melanopic information and actual exposure depends strongly on distance, gaze direction, room geometry, and daylight availability [14].

### Current policy approaches

Several public health and standards organizations have contributed to the evidence base and to early translation. France’s national health agency ANSES has examined health risks related to LED lighting and blue-light exposure. The UK Health Security Agency contributed to the development of CIE S 026/E:2018, which provides a system for measuring ipRGC-influenced responses to light [13]. The international expert recommendations by Brown [2] provide quantitative targets for daytime, evening, and nighttime indoor light exposure in healthy adults. Germany’s Federal Institute for Occupational Safety and Health is examining real-world effects of light exposure in work settings. These efforts show that public health organizations are already engaged, but guidance remains uneven and often technical, hazard-focused, or narrow in scope.

### Communication challenges and limits

Population-wide guidance is appropriate when it is framed as a baseline, low-risk direction of travel rather than as individualized dosing. People differ in light sensitivity, prior light exposure, chronotype, age, ocular media, pupil size, genetics, behavior, health status, work schedules, and living environments [15, 16]. Older adults may need more light to achieve similar retinal stimulation because age-related optical changes reduce light transmission to the retina [17]. Shift workers, people with circadian rhythm sleep-wake disorders, people with some eye conditions, infants, and some clinical populations may require tailored advice.

Sunlight messaging also requires care. Public health communication should continue to emphasize protection against excessive ultraviolet exposure and should not present ultraviolet exposure as a circadian intervention. At the same time, daylight is often the most accessible way to obtain high visible-light exposure during the day. These messages can coexist: people can seek daylight while using shade, clothing, sunglasses, and sunscreen when appropriate. The goal is visible light at the eye during the day, not unsafe ultraviolet exposure.

Geography and season shape implementation. In high-latitude regions, winter messaging may need to emphasize bright indoor environments and outdoor exposure when available, while summer messaging may need to address late-evening daylight and bedroom darkness. In hot climates, advice to spend time outdoors should be adapted to heat, air quality, safety, and local norms. In all settings, recommendations should be equitable and should acknowledge that people do not have equal control over housing, work schedules, school environments, transport, or access to outdoor space.

## Policy options

Public health organizations can act at three levels: communication, environments, and evidence infrastructure. These options are complementary rather than mutually exclusive.

### Option 1: communicate a simple 24-h light pattern

Public health organizations should make the central public message explicit: seek brighter light during the morning and daytime, reduce bright light during the evening, and sleep in darkness or near-darkness at night. This phrasing is more precise than “dim light in the evening and at night” because evening and sleep periods have different practical goals. Evening light can often be reduced rather than eliminated; the sleep environment should be as dark as feasible.

Actionable public-facing tips can include the following:

- Spend time in daylight each day, especially within the first few hours after waking, while following local sun-safety advice.
- When indoors during the day, sit near windows where feasible and use sufficiently bright indoor lighting when daylight is unavailable.
- In the three hours before bedtime, dim electric lights, avoid very bright screens close to the eyes, or use settings that reduce short-wavelength light when screens are necessary.
- Keep the sleep environment dark or near-dark by limiting light leaks, using curtains or masks where acceptable, and avoiding unnecessary overnight lighting.
- Use night lights only when needed for safety, and keep them low, shielded, and away from direct view.

These messages should avoid unsupported precision. Current evidence does not justify a universal instruction such as “X minutes of bright light per day” for all people. A lunchtime walk or morning outdoor time may be helpful for many indoor workers, but public guidance should present this as a practical example rather than a fixed prescription.

### Option 2: integrate light into health-promoting environments

Individual behavior change is limited by the environments in which people live, work, study, and receive care. Public health organizations should therefore encourage settings-based implementation. Workplaces and schools can prioritize access to daylight and adequate daytime indoor light, especially in the morning. Healthcare and elder-care settings can consider brighter daytime light, reduced evening light, and protected nighttime darkness, while maintaining safety, mobility, and clinical needs. Housing guidance can address daylight access, bedroom darkness, and practical strategies for evening lighting.

Implementation should avoid simplistic product recommendations. Lamp labels such as lumens, correlated color temperature, wattage, energy efficiency, or “LED” do not reliably indicate the non-visual stimulus at the eye. Where technical guidance is needed, melanopic EDI is more relevant than conventional photopic illuminance alone. However, because melanopic values are not routinely provided to consumers, daylight access and measured *in situ* exposure remain more reliable than product categories for general guidance.

### Option 3: support tailored guidance, research, and labeling

Public health organizations should support research that turns the general principle into more specific guidance for different groups and contexts. Priorities include the duration and timing of daytime light needed for different outcomes, interactions between daytime and evening exposure, responses in children and older adults, guidance for shift workers, implementation in schools and healthcare settings, and equitable strategies for people with limited control over their environments.

Organizations should also support practical measurement and labeling. Wider availability of melanopic quantities on lighting products and in lighting assessments would make it easier for professionals and eventually consumers to understand whether an environment is likely to provide sufficient daytime stimulation or excessive evening and nighttime stimulation. Any labeling effort should communicate that real exposure still depends on distance, direction, duration, and context.

### Recommended approach

The recommended approach is a staged policy package. First, publish plain-language guidance centered on bright days, dim evenings, and dark or near-dark nights. Second, align that guidance with sun-safety messaging so that visible daylight exposure and ultraviolet protection are presented together rather than as competing priorities. Third, embed light exposure considerations into settings where individual choice is limited, including workplaces, schools, healthcare, elder care, and housing. Fourth, support research and labeling efforts that can move future guidance from general principles toward more precise and individualized recommendations.

This package gives public health organizations a clear message to communicate now, while acknowledging the limits of the current evidence and the need for tailored advice in special circumstances.

## Conclusion

Light is a modifiable environmental exposure with relevance for circadian alignment, sleep, mood, alertness, and cognition. Public health organizations can translate current evidence into practical guidance without overstating precision: brighter light during the morning and daytime, reduced light in the evening, and darkness or near-darkness during sleep. This message should be paired with sun-safety advice, adapted for geography and setting, and presented as general guidance rather than individualized treatment. By combining clear communication, healthier environments, and support for research and labeling, public health organizations can help people align daily light exposure with human physiology while preserving flexibility for individual needs.
